# Relationship Between Obesity and Impairment of Cognitive Functions: An Investigation into the Integrated Role of Nutritional Education and Physical Activity in Lower Secondary School

**DOI:** 10.3390/nu17152531

**Published:** 2025-07-31

**Authors:** Maria Giovanna Tafuri, Domenico Tafuri, Francesca Latino

**Affiliations:** 1 Department of Literary, Linguistic and Philosophical Studies, Pegaso University, 80143 Naples, Italy; 2 Department of Medical, Motor and Wellness Sciences, University of Naples “Parthenope”, 80143 Naples, Italy; 3 Department of Education and Sport Sciences, Pegaso University, 80143 Naples, Italy

**Keywords:** executive functions, school-based intervention, cognitive performance, health promotion, motor skills, school-aged children

## Abstract

Background/Objectives: Obesity in adolescence is associated with a deterioration in cognitive functions, with significant implications for psychophysical well-being and academic performance. Recent studies highlight the importance of integrated interventions that combine nutrition education and physical activity to promote the overall health of students. The present study aims to evaluate the efficacy of an integrated intervention based on nutritional education and conscious body movement in improving cognitive functions, perceived well-being and nutritional knowledge in lower secondary school students with indicators of overweight and obesity. Methods: A quasi-experimental design with randomization at the class level was adopted, involving 60 students divided into an experimental group and control group. The intervention was divided into twelve weeks of activities, divided between nutritional education modules and physical activity courses. Standardized tests for the assessment of cognitive functions (Digit Span Forward, Digit Span Backward, Stroop Test, Trail Making Test B), motor tests (6-Minute Walk Test, Sit and Reach Test) and a food knowledge questionnaire were administered before and after the intervention. Results: The experimental group showed significant improvements compared to the control group in all cognitive, motor, and nutritional knowledge measures, indicating the effectiveness of the integrated intervention in promoting cognitive and physical well-being. Conclusions: The findings support the role of school as a generative environment of integrated well-being, suggesting the need to develop and implement curricular programs that integrate nutrition education and physical activity to counteract the negative effects of obesity on cognitive function in adolescents.

## 1. Introduction

The increase in childhood obesity represents one of the most relevant emergencies globally, configuring itself not only as a physical health problem but also as a risk factor for the cognitive, emotional and social development of children and adolescents [1]. According to data from the World Health Organization [2], obesity in childhood has reached alarming proportions in many countries, including middle-high income countries such as Italy, where there is a growing incidence especially in the age group between 11 and 14 years [3], with recent national data showing that 20.1% of children aged 8–9 are overweight and 9.8% are obese and with higher rates observed in South Italy [4]. These rates tend to increase in early adolescence. Furthermore, physical activity levels are critically low in this age group: only 9.5% of Italian students aged 11–14 meet WHO recommendations for daily physical activity, while over 18% spend more than 3 h a day displaying sedentary behavior, including screen time [5]. This phenomenon, in addition to being a known risk factor for numerous chronic diseases, has a significant correlation with the alteration of cognitive development and with the impairment of some executive functions fundamental for school learning, such as working memory, sustained attention, planning and behavioral self-regulation [6], as also confirmed by studies that identify significant deficits in executive functioning among children with obesity, particularly in inhibitory control, cognitive flexibility and attention maintenance [7,8].

The picture emerging from the scientific literature therefore underlines the urgency of preventive and educational interventions capable of acting in an integrated way, addressing not only the nutritional and motor aspects, but also the affective and relational dimensions of body well-being. In particular, some neuroimaging studies have documented alterations in the connectivity of brain areas involved in decision-making processes and emotional regulation, with a negative impact on both eating behavior and the ability to maintain adequate attention and concentration during school activities [9]. In addition, obesity is frequently associated with a condition of poor body perception, social isolation and low self-esteem—all elements that, interacting with cognitive vulnerabilities, can significantly compromise the overall well-being of the student.

In this context, school plays a crucial role as a privileged environment for the promotion of health and food education, as it represents the space of daily life of young people, where habits are built, learning is consolidated and social skills are developed [10]. The relevance of the school context is not limited to the transmission of information but extends to the possibility of activating complex educational processes, capable of affecting the lifestyle and conscious choices of children. However, traditional approaches to nutrition education and physical activity, often based on prescriptive models and functional logic, show significant limitations [11]. Nutritional education is frequently confined to transmitting theoretical content and behavioral advice, while motor activity is reduced to moments of prescriptive motor activity aimed at movement as a mere anti-stress or physical “discharge”, without an effective ability to deeply affect the cognitive, affective and motivational mechanisms that support the daily habits of pre-adolescents [12,13].

The growing body of literature emphasizes the need for school-based interventions that simultaneously address the motor, cognitive, and emotional dimensions of students’ development. Recent studies have shown that integrated programs combining physical activity with nutrition education contribute to significant improvements not only in BMI and eating habits, but also in cognitive outcomes such as attention, self-regulation and academic performance [14].

On the basis of these considerations, it is essential to explore educational pathways that can promote both cognitive enhancement and lifestyle improvement among students, avoiding disciplinary fragmentation. The theoretical framework of the intervention is based on recent acquisitions in the neuroscientific field that relate motor activity and cognitive functioning. It is now widely documented that physical activity, especially if carried out in a conscious and non-competitive way, promotes the release of neurotransmitters and neurotrophins, including BDNF (Brain-Derived Neurotrophic Factor), which play a crucial role in synaptic plasticity and neurogenesis [15]. The increased cerebral blood flow induced by movement, together with sensory and proprioceptive stimulation, helps to strengthen the neuronal circuits involved in complex cognitive tasks, improving students’ executive performance. In addition, structured body movement has been shown to positively affect mood, stress management skills and perceived self-efficacy, all of which, in turn, influence motivation to learn and willingness to change one’s behaviours [16]. At the same time, nutritional education is declined here in a critical and dialogical perspective, which goes beyond the mere prescription of dietary rules to open up to a reflection on the representations, emotions and socio-cultural influences that act on food choices. This approach aims to strengthen students’ metacognitive skills, encouraging them to question their habits and activate more conscious decision-making processes, supported by an understanding of the interactions between food, body and mind [17]. Also in this case, the cognitive dimension is placed at the center of educational action, since a balanced diet cannot be imposed from the outside, but must emerge as the result of an autonomous re-elaboration of knowledge and experiences [18].

Numerous national and international studies support the effectiveness of integrated interventions that combine nutritional education and physical activity in the school context [19,20,21]. They document the effectiveness of integrated school–nutrition–movement interventions, highlighting how programs that combine nutritional education and structured and conscious physical activity lead to better outcomes than one-dimensional interventions [22]. Empirical evidence shows that such approaches not only reduce the incidence of childhood obesity, but also produce significant improvements in cognitive performance and academic performance, especially in the domains of sustained attention and cognitive flexibility [23]. In particular, the integration of structured, creative and environmental bodily experiences makes it possible to activate embodied learning processes, in which knowledge is no longer separated from the body, but passes through it, in a continuous dialogue between perception, emotion and cognition [24].

The present article contributes to this line of research by proposing an integrated school-based intervention and exploring its cognitive and educational implications. Building on the theoretical and empirical premises discussed above, the study aims to investigate how a combined approach to nutritional education and conscious movement can promote both health and executive function development among lower secondary school students. Rather than relying on prescriptive or isolated strategies, the intervention adopts a holistic and participatory framework, grounded in neuroscientific, pedagogical, and embodied learning perspectives.

In this way, this work seeks to enrich the scientific discourse on how schools can respond in an innovative and interdisciplinary way to the complex challenges posed by childhood obesity. It emphasizes the central role of educational action in shaping not only visible behaviors but also the underlying cognitive and motivational structures from which those behaviors emerge. Only through integrated, reflective, and student-centered interventions is it possible to promote lasting transformations in lifestyle, self-regulation, and well-being, recognizing the inseparable link between body, nutrition, and mind.

## 2. Materials and Methods

### 2.1. Study Design

The present study adopts a quasi-experimental design [25] with an experimental and a control group, aimed at evaluating the effectiveness of an integrated intervention centered on nutritional education and conscious, creative, and environmental body movement in promoting improvements in cognitive functions in lower secondary school students with indicators of overweight and obesity. The investigation is part of applied educational research, taking as its theoretical–methodological framework the action–research approach, which combines transformative purposes with the rigor of empirical monitoring.

A non-probability, purposive sampling procedure was employed to select the participating school and classes. The school was chosen based on a direct request from its administration, which reported a high prevalence of overweight and obesity among its students. This allowed for the identification of a population particularly suitable for the proposed intervention. Group assignment was performed at the class level, using random allocation among the available classes, avoiding the fragmentation of existing classroom groups and maintaining organizational coherence. The quasi-experimental structure preserved ecological validity by operating in a real school setting while ensuring group comparability through the standardization of evaluation procedures and sample homogeneity.

The study was conducted over three phases: a preliminary phase for sample selection and baseline data collection (pre-test), an implementation phase of the educational intervention, and a final evaluation phase (post-test). The intervention was carried out over a twelve-week period during ordinary school hours, with the involvement of curricular teachers and external experts. The activities were transversally integrated into the educational offer plan and aligned with the school’s educational objectives. The experimental group participated in the integrated program, while the control group continued with standard curricular activities without substantial changes related to physical education or health.

Data were subjected to statistical analysis to identify significant differences between groups in the targeted cognitive domains, with particular attention to executive functions, selective attention, and working memory.

The study was approved by the Department of Medical, Motor, and Wellness Sciences at the University of Naples “Parthenope” (DiSMMeB Prot. N. 88592/2024) and conducted in accordance with the ethical principles of the Declaration of Helsinki. Informed consent was obtained from the families of all participants.

### 2.2. Participants

The sample (Table 1) consisted of sixty students enrolled in the second year of lower secondary school, aged between twelve and thirteen, from a medium-sized urban school in southern Italy. The selection criteria followed a purposive sampling of the school and a random allocation of classes to the experimental or control condition. Participants included in the study were students enrolled in the second year of lower secondary school, typically aged between 12 and 13 years. Only those with regular school attendance were considered eligible, as consistent participation was essential for the effectiveness of the intervention. In addition, inclusion required that informed consent had been obtained, signed by the students’ parents or legal guardians in accordance with ethical research standards. Participants were excluded from the study if informed consent had not been obtained. They were also excluded if they presented acute medical, psychological, or motor conditions that, based on clinical or school-based assessments, would have made it unsafe or unfeasible for them to participate in the proposed activities. This exclusion was not related to the presence of a disability per se. In fact, students with disabilities were actively included in the study, provided that their participation was compatible with the intervention, and appropriate methodological, educational, and organizational accommodations were put in place to support their full and meaningful engagement in line with principles of equity and educational inclusion.

Anthropometric data were collected in the preliminary phase by specialized personnel according to WHO guidelines [26]. Overweight and obesity conditions were identified via BMI measurements (weight and height). Among the participants, 36% in the experimental group and 34% in the control group had BMI values above the age-specific thresholds, ensuring comparability between groups in the variable of interest.

The experimental group (*n* = 30) participated in the integrated intervention combining nutritional education and structured body movement modules. The control group (*n* = 30) continued the usual school program. The final composition of the groups reflected age, gender, and BMI homogeneity, which was confirmed through the preliminary data collection.

Although the absolute number of participants was limited, the sample size was adequate for statistical analyses, due to the rigor of the research design and the consistency of the data collection process. Teachers actively collaborated in the study, particularly in facilitating participation and ensuring adherence to the intervention, which contributed to the ecological validity of the research.

### 2.3. Procedures

**Figure 1:**
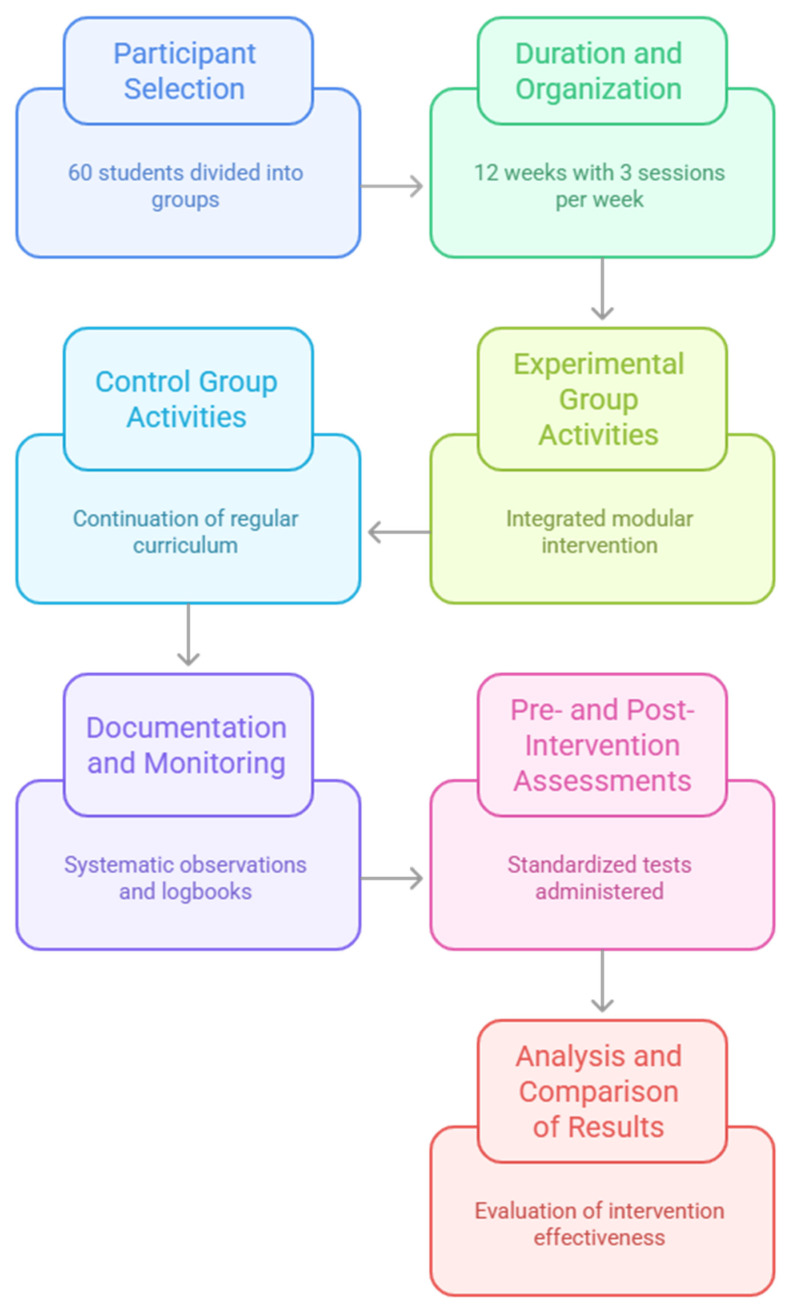
Flowchart of the study.

The intervention was carried out over a twelve-week period and was integrated into the regular school timetable (Figure 1). Sessions were held three times per week, each lasting sixty minutes, for a total of thirty-six meetings. All activities took place in commonly used school settings, such as classrooms, gyms, and courtyards and, whenever feasible, extended to nearby outdoor spaces like public gardens or urban green areas. The experimental group participated in the planned sessions, while the control group continued with the standard educational program as outlined in the school curriculum.

The implementation of the activities was coordinated with the teaching staff and approved by the class councils to ensure alignment with the school’s educational goals. All procedures were conducted in accordance with established ethical and safety standards to ensure participant well-being and data integrity. Participants wore suitable clothing to allow safe and effective participation.

Throughout the intervention, the research team documented each session through structured observations and reflective logbooks, in order to monitor attendance, engagement levels, and the overall quality of the educational interactions. These instruments were not subjected to a systematic qualitative analysis but were employed solely to support the data collection process and to ensure the consistency and reliability of the quantitative measurements. Standardized assessments were administered to both the experimental and control groups before and after the intervention under equivalent conditions, in order to evaluate outcomes related to cognitive functioning, well-being, and nutritional awareness. The approach adopted aimed to ensure continuity between the intervention and the broader educational experience, recognizing the school context as a key environment for the holistic development and health promotion of all students.

### 2.4. Experimental Intervention

The experimental intervention (Table 2) was structured as an intensive course lasting a total of twelve weeks, divided into thirty-six meetings, with a frequency of three weekly sessions of sixty minutes each. The general objective was to explore the combined effect of a nutritional education and structured motor activity program on cognitive functioning, psychophysical well-being and some health-related parameters in lower secondary school students. The intervention was designed to integrate content and practices capable of activating neurophysiological mechanisms favorable to cognitive development, emotional regulation and prevention of conditions associated with childhood obesity, with particular attention to executive function, working memory and cognitive flexibility.

The experimental group participated in the entire course within school hours, while the control group followed the regular curriculum, without significant changes in the teaching routine.

*Nutritional Education Intervention*

The nutritional module included twelve meetings conducted by a team made up of a clinical nutritionist and a science teacher, with the methodological support of an educational researcher. The focus of the intervention was oriented towards the development of knowledge and skills useful for understanding the role of nutrition in supporting brain function, concentration, memory and metabolic regulation.

Specific topics related to energy requirements in developmental age, the glycemic index and its influence on attention levels, macronutrient composition in relation to cognitive performance and daily eating habits that interfere with neurophysiological rhythms were discussed. The mechanisms of correlation between excess weight, chronic low-grade systemic inflammation and decline in executive functions were analyzed. In parallel, students were offered the opportunity to acquire practical tools to assess food quality, plan balanced meals and reflect on the environmental and social determinants of food choices.

The teaching methodology included interactive activities, simulations, cognitive games, analysis of nutritional labels, self-monitoring through food diaries and discussion of simplified clinical cases. The approach focused on the connection between theory and practice, with the aim of strengthening the role of nutrition education in promoting cognitive health and energy regulation.

*Physical Activity Intervention*

At the same time, the motor module was divided into twenty-four meetings conducted by specialists in motor science with specific training in exercise neurophysiology. The intervention was designed according to carefully calibrated progression to stimulate the main components of neuro-motor efficiency, with expected effects on the executive, attentional and mnemonic levels.

The activities were divided into three phases. The first included low-impact exercises focused on improving postural awareness and diaphragmatic breathing, with the aim of promoting emotional self-regulation and reducing stress, recognized as an interfering factor in cognitive processes. The second phase introduced moderate coordinative and rhythmic motor activities, including varied locomotion exercises, open-structure games and dynamic circuits that required the use of working memory and rapid decision-making. Finally, the third phase involved outdoor motor experiences in urban and natural contexts, with brisk walking, spatial orientation activities and cognitive tasks associated with movement (dual-task training), with the aim of stimulating neuroplasticity through multisensory integration. The sessions were conducted in small groups.

Overall, the integrated intervention aimed to determine a positive impact on the neurocognitive functioning of overweight and obese students, through the synergy between the acquisition of more balanced eating behaviors and the activation of body pathways aimed at neuroendocrine regulation and the improvement of executive functions.

**Table 2:** Weekly structure of the integrated intervention.

Week	Nutritional Education (1 Weekly Session)	Physical Activity (2 Weekly Sessions)
1	Introduction to the relationship between nutrition and the brain: how food influences mental performance	Basic body awareness: posture, breathing, relaxation
2	Macronutrients and cognitive functions: focus on carbohydrates, proteins, fats	Dynamic postural education and breath control
3	Glycemic index, attention and memory: effects of glycemic fluctuations	Balance exercises, motor control and spatiotemporal perception
4	Physiological vs. emotional hunger: self-regulation and bodily signals	Simple coordination paths and progressively challenging games
5	Nutrition labels: reading and critical interpretation	Rhythmic activities, attention and reaction games, motor memory tasks
6	Breakfast and school meals: impact on cognitive performance	Dual-task exercises: integration of motor tasks and cognitive challenges
7	Food and concentration: brain-friendly nutrients	Group motor paths with cooperative and decision-making tasks
8	Sugary drinks and attention decline	Outdoor activities: paced walking and environmental observation
9	Inflammation and obesity: effects on the brain and behavior	Spatial orientation games and environmental recognition
10	Building a balanced meal: nutritional logic and sustainability	Narrative motor activities in outdoor settings: movement and storytelling
11	Food stereotypes and social influences on eating choices	Multisensory motor experiences: bodily exploration of natural environments
12	Final reflection and self-assessment: toward conscious food choices	Integrative activities: review of motor and cognitive learning outcomes

### 2.5. Measures

The evaluation of the effectiveness of the intervention was conducted through an integrated set of standardized and validated tools, capable of measuring both cognitive functions potentially influenced by obesity and physical activity, and some motor and physiological components relevant to the overall psychophysical well-being of the student. In addition, tests were used to detect changes in the nutritional domain. Measurements were made at two times: before the start of the program (pre-test) and at the end of the twelve-week cycle (post-test), according to a longitudinal design with comparison between experimental and control groups.

*Assessment of cognitive functions*

The selection of cognitive tests followed the criterion of sensitivity to variations associated with nutritional and motor practices, with particular attention to executive functions, working memory, cognitive flexibility and attentional capacity.

- **Digit Span Test—Forward and Backward (Wechsler Intelligence Scale for Children, WISC-IV)**
  The Digit Span Test [27] was used to assess working memory and short-term attention. In the Forward version, the student must repeat a sequence of digits in the order presented, while in the Backward version, the student is required to repeat the digits in reverse order. Increasing the length of the sequence allows you to estimate the ability to actively maintain and manipulate information in memory. The Digit Span Test, both Forward and Backward, was used to assess working memory capacity. Previous research has reported acceptable internal consistency, with Cronbach’s alpha values ranging from 0.70 to 0.80 in school-aged children [28].
- **Stroop Color and Word Test—Children’s Version**
  The Stroop Color and Word Test [29] is a well-established tool for measuring inhibitory control and cognitive flexibility. Participants are asked to name the color of the ink while ignoring the semantic meaning of the written word. The test allows us to highlight the efficiency of the processes of automatic inhibition, switching and selective attention, considered sensitive markers of executive efficiency in developmental age. The Children’s Version of the Stroop Color and Word Test was employed to measure selective attention and cognitive inhibition. The test demonstrates satisfactory reliability, with Cronbach’s alpha values typically reported between 0.75 and 0.85 [30].
- **Trail Making Test—Parts A and B (version adapted for school age)**
  The Trail Making Test [31] assesses processing speed, the ability to toggle between different cognitive sets (letters and numbers), and the executive organization of behavior. The completion times of the two parts of the test, as well as the errors made, offer useful indicators of the level of cognitive flexibility and visuospatial planning. The adapted school-age version of the Trail Making Test (Parts A and B) was used to evaluate cognitive flexibility and processing speed. Test–retest reliability for children has been reported as good, with ICC values around 0.80 [32].
- **Raven’s Progressive Matrices Test—Coloured Progressive Matrices (CPMs)**
  Included as a measure of nonverbal logical reasoning and the ability to solve abstract visual problems, the Raven test [33] also allows us to detect any general differences in intellectual functioning between groups, controlling for any pre-existing cognitive biases. Raven’s Coloured Progressive Matrices (CPMs) were administered to assess non-verbal fluid intelligence. The internal consistency of the CPM is high, with Cronbach’s alpha typically above 0.85 [34].

*Assessment of motor and functional condition*

In parallel, simple yet reliable tools were adopted to assess physical and motor efficiency, with particular attention to aspects related to coordination, endurance, and body composition, in line with the physiological targets of the intervention.

- **6-Minute Walk Test (6MWT)**
  The 6-Minute Walk Test [35] was used to estimate cardiorespiratory capacity and functional aerobic endurance. The test requires participants to cover the maximum possible distance in six minutes on a straight track. It was chosen for its validity in school contexts and sensitivity to changes related to regular physical activity. The 6-Minute Walk Test (6MWT) was used to evaluate submaximal aerobic capacity and functional endurance. Reliability studies in children report high test–retest reliability with ICC values ranging from 0.90 to 0.95 [36].
- **Sit and Reach Test**
  The Sit and Reach Test [37] measures the flexibility of the lower back and hamstrings. Although not directly related to cognitive functions, it represents a general index of muscle tone and joint mobility, influenced by the level of sedentary lifestyle. The Sit and Reach Test was administered to assess hamstring and lower back flexibility. It is a widely used field test with good test–retest reliability, with ICC values typically between 0.85 and 0.92 in children [38].

*Assessment of anthropometric parameters*

- **Body Mass Index (BMI)**
  According to the criteria defined by Cole in 2002 [39], the body mass index (BMI) was calculated for each participant using the weight divided by height squared formula, expressed in kilograms per square meter (kg/m^2^), and interpreted with reference to internationally validated age- and sex-specific cut-offs for the pediatric population. This indicator was used both as a measure of weight status and as a continuous variable in the analysis of pre- and post-intervention variations, with the aim of detecting any changes in the body profile attributable to educational and motor interventions. Body Mass Index (BMI) was calculated using standardized procedures. Anthropometric measurements were conducted by trained personnel, and BMI demonstrates high inter-rater reliability, with ICC values exceeding 0.95 [40].
- **Waist Circumference**
  Abdominal circumference [41] was measured at the height of the navel with millimeter tape, as an additional indicator of metabolic risk and central fat accumulation, as it is more related to neuroinflammatory risks than BMI alone. Waist circumference was measured using a non-elastic tape at the midpoint between the last rib and the iliac crest. This measure has shown excellent inter-rater reliability in pediatric populations, with ICC values above 0.90 [42].

*Assessment of Nutritional Intervention*

- **Questionnaire on nutritional knowledge (adapted from Parmenter & Wardle, 1999 [43])**
  We used a simplified version of this questionnaire adapted for school age [43], aimed at verifying the understanding of fundamental concepts related to food groups, energy requirements, meal balance and reading nutrition labels. It includes multiple choice and true/false questions, structured in thematic sections. The overall score makes it possible to quantify the effectiveness of the educational path in terms of knowledge acquisition. Nutritional knowledge was assessed using a questionnaire adapted from Parmenter and Wardle [43], which has demonstrated good internal consistency in various populations, with reported Cronbach’s alpha values around 0.78.

All tests were administered by trained operators, according to standardized protocols, in quiet school environments and under controlled environmental conditions. The tools were administered in paper form, with the supervision of the teacher and assistance from the research team, to ensure linguistic comprehension and limit any bias related to the level of literacy. The order of administration was kept constant between pre- and post-intervention to reduce the effect of confounding variables. The data were subsequently coded and subjected to statistical analysis to evaluate the effectiveness of the intervention on a comparative basis between the two groups.

### 2.6. Statistical Analysis

The data processing was carried out using IBM SPSS Statistics software (version 25.0; IBM Corp., Armonk, NY, USA), adopting a significance level of *p* < 0.05. The aim of the analysis was to verify the effectiveness of the integrated intervention, comprising nutritional education and conscious bodily activity, in improving cognitive functions, nutritional status, and motor efficiency in lower secondary school students.

In the first phase, a descriptive analysis of the variables collected at pre- and post-intervention was conducted, reporting for each the mean (M), standard deviation (SD), minimum and maximum values, and percentage distribution of nominal categories. A confirmatory factor analysis (CFA) was then performed to evaluate the construct validity of the measurement instruments. This analysis aimed to verify the latent structure underlying each psychometric and physiological instrument, and to assess the theoretical coherence of the measured constructs in relation to the intervention’s objectives.

To ensure initial comparability between the experimental and control groups, the Chi-square test was used for categorical variables.

In order to assess the effectiveness of the intervention while accounting for initial differences between groups, an Analysis of Covariance (ANCOVA) was conducted for each dependent variable (cognitive functions, physical abilities, and nutritional knowledge), with the group (experimental vs. control) as the between-subject factor and the pre-test scores as covariates. This method allowed for statistical control of baseline variability and a more precise estimation of the intervention’s effect. The assumptions of ANCOVA, including homogeneity of regression slopes and normal distribution of residuals, were checked and met. No data transformations were applied, as the residuals displayed acceptable normality, allowing for the preservation of the original scale of measurement and interpretability of results. Effect sizes were calculated using partial eta squared (η^2^_p_), which quantifies the proportion of variance explained by the group factor after controlling for covariates. According to Richardson [44], values of η^2^_p_ between 0.00 and 0.009 are considered negligible, 0.01–0.059 small, 0.06–0.139 medium, and ≥0.14 large. 

Correlation analysis was also performed using Pearson’s correlation coefficient (r) to explore the linear relationships between changes in the main outcome variables. In particular, the associations between improvements in cognitive performance and changes in anthropometric parameters (BMI, waist circumference), nutritional scores (KIDMED), and motor performance indices (6MWT) were examined.

Finally, potential confounding variables, including age, sex, and the initial score on the Raven Progressive Matrices, were included as covariates in the multivariate models to control for the influence of exogenous factors on the variation in the dependent variables.

## 3. Results

### 3.1. Confirmatory Factor Analysis and Construct Validity of the Measurement Scales

The CFA (Table 3) was conducted using the Maximum Likelihood Estimation method, after verification of multivariate normality and sample adequacy by means of the KMO (Kaiser–Meyer–Olkin) index and the Bartlett sphericity test. The expected values for an adequate CFA are KMO > 0.70, significance of the Bartlett test (*p* < 0.05), non-significant chi-square or with a χ^2^/*df* ratio < 3, CFI (Comparative Fit Index) and TLI (Tucker–Lewis Index) values ≥ 0.90, RMSEA (Root Mean Square Error of Approximation) ≤ 0.08.

The data collected through the standardized scales were aggregated into three theoretical latent domains: cognitive functions, assessed using the Digit Span Forward and Backward, the Stroop Color and Word Test, the Trail Making Test parts A and B, and the Raven’s Coloured Progressive Matrices; physical and anthropometric condition, measured through the 6-Minute Walk Test (6MWT), the Sit and Reach Test, Body Mass Index (BMI), and waist circumference; and nutritional knowledge, evaluated using a questionnaire adapted from Parmenter and Wardle (1999) [43].

The CFA confirmed a three-factor model with adequate internal consistency and good adherence to empirical data. The following indicators support the goodness of the model:

1. Overall KMO = 0.81;
2. Bartlett’s test: χ^2^(36) = 228.54, *p* < 0.001;
3. χ^2^/*df* = 1.89;
4. CFI = 0.95;
5. TLI = 0.93;
6. RMSEA = 0.064, 90% CI [0.048, 0.078];
7. SRMR = 0.051.

All standardized factor loads were statistically significant (*p* < 0.001) and greater than 0.50, with values ranging from 0.58 (Sit and Reach Test) to 0.84 (Digit Span Backward), indicating good saturation of theoretical factors.

### 3.2. Descriptive Statistics of Pre- and Post-Test Measurements

Table 4 summarizes the means and standard deviations of the key outcome variables measured before and after the intervention for both the experimental and control groups. The table includes cognitive function scores, physical performance indicators, nutritional knowledge, and anthropometric parameters such as BMI, weight, and waist circumference. Additionally, the mean change (Δ) from pre- to post-test is reported to highlight the magnitude and direction of improvement or decline within each group. These descriptive statistics provide an essential overview of the data prior to the inferential analysis presented in the subsequent ANCOVA.

### 3.3. ANCOVA

Considering the nature of the quasi-experimental design with pre- and post-intervention measurements and the need to control for initial differences between the experimental and control groups, an analysis of covariance (ANCOVA) was conducted, which allows us to compare post-intervention scores between groups, controlling for pre-intervention scores as a covariate (Table 5). The ANCOVA was performed for each dependent variable (cognitive functions, physical capacity, nutritional knowledge), with the group (experimental vs. control) as the independent variable and the pre-test score as the covariate. The results showed significant group principal effects in favor of the experimental group in all measures considered, even after controlling for initial scores. In particular, for the Digit Span Forward, the effect of the group was significant, where F(1,57) = 14.86, *p* < 0.001, η^2^ = 0.21, indicating that the experimental group maintained a substantial advantage over the control, net of initial differences. Similarly, in the Digit Span Backward, a significant group effect is observed, where F(1,57) = 12.45, *p* = 0.001, η^2^ = 0.18. For the Stroop Test, the corrected response times for the pre-test are significantly lower in the experimental group, where F(1,57) = 16.92, *p* < 0.001, η^2^ = 0.23, and also for the Trail Making Test—Part B, a significant effect is observed, where F(1,57) = 11.08, *p* = 0.001, η^2^ = 0.16. In the motor setting, the 6-Minute Walk Test showed a significant group effect, where F(1,57) = 13.74, *p* < 0.001, η^2^ = 0.19, and the Sit and Reach Test confirmed a post-intervention improvement in favor of the experimental group, where F(1,57) = 10.33, *p* = 0.002, η^2^ = 0.15. ANCOVA analysis, conducted by controlling for pre-test values, confirmed a significant effect of motor and educational intervention on BMI [F(1,57) = 6.4, *p* = 0.05, η^2^ = 0.10], waist circumference [F(1,57) = 8.3, *p* = 0.01, η^2^ = 0.12], and Weight [F(1,57) = 7.10, *p* = 0.01, η^2^ = 0.11]. These results suggest that the observed reduction in post-test values can be attributed to the intervention, regardless of initial differences between groups. Finally, nutritional knowledge showed a marked difference even after the control for pre-test scores, where F(1,57) = 18.60, *p* < 0.001, η^2^ = 0.25. Overall, these results confirm the effectiveness of the educational and motor intervention in significantly improving cognitive, physical, and behavioral indicators, also considering the initial conditions of the participants, with effects of medium to high magnitude (η^2^ from 0.10 to 0.25) in favor of the experimental group.

**Figure 2:**
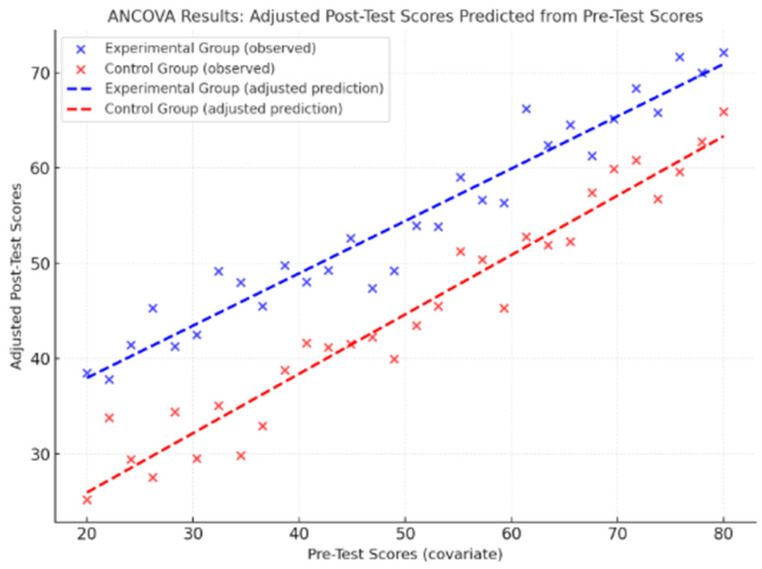
Results of the ANCOVA analysis. The scatterplot illustrates the relationship between pre-test scores (covariate) and adjusted post-test scores for the experimental group (blue) and the control group (red). The dotted regression lines represent the adjusted predictions, showing that for equivalent pre-test scores, participants in the experimental group achieved significantly higher post-test results compared to those in the control group.

The following graph represents the ANCOVA analysis showing the relationship between the pre-test scores (covariate) and the adjusted post-test scores for the experimental group (in blue) and the control group (in red) (Figure 2). The dotted lines indicate the regression lines derived from the ANCOVA, highlighting that, with the same initial score, the experimental group obtained significantly higher adjusted post-intervention results than the control group, visually confirming the effectiveness of the integrated intervention.

### 3.4. Pearson Correlation

To explore the relationships between cognitive, physical, and nutritional variables in the experimental group after the intervention, an analysis of Pearson’s correlation on post-test scores was conducted (Table 6). The results indicate positive and statistically significant associations between cognitive functions and physical performance, suggesting a synergistic relationship between cognitive improvements and increased motor capacity. In particular, the score on the Digit Span Forward was positively correlated with the 6-Minute Walk Test (r = 0.52, *p* = 0.003) and with the nutritional knowledge score (r = 0.49, *p* = 0.005), indicating that greater working memory is associated with better physical endurance and higher food proficiency. Similarly, the Digit Span Backward showed significant correlations with the Sit and Reach Test (r = 0.46, *p* = 0.008) and nutritional knowledge (r = 0.50, *p* = 0.004), suggesting that working memory is positively associated with both motor flexibility and food awareness. Trail Making Test B is negatively correlated (expected value, since shorter times indicate better performance) with the 6MWT (r = −0.55, *p* = 0.002) and positively with the Digit Span Backward (r = −0.48, *p* = 0.006), highlighting that better shifting capacity and sustained attention is associated with better physical and operational memory performance. Correlation analyses show a significant pattern between cognitive abilities, nutritional skills and fitness indicators. The results reveal positive correlations between memory skills (Digit Span) and physical performance (6-Minute Walk Test and Sit and Reach); executive efficiency (Trail Making Test B) is negatively correlated with memory skills and physical performance, consistent with the idea that better cognitive performance is associated with shorter times in the TMT-B test; BMI and waist circumference show moderate and negative correlations with fitness tests and nutritional knowledge. A higher BMI is associated with lower performance on the walk test, less flexibility, and less food knowledge. In particular, waist circumference has slightly stronger correlations than BMI, highlighting its role as a more sensitive marker of cardiometabolic risk in developmental age.

Finally, nutritional knowledge shows strong correlations with all the cognitive variables considered, in particular with the Digit Span Forward (r = 0.49, *p* = 0.005) and the Trail Making Test B (r = −0.51, *p* = 0.004), underlining the transversal role of food awareness in cognitive processes. These results support the hypothesis that an integrated approach on the educational and motor level can produce synergistic effects in the overall improvement of school functioning.

**Figure 3:**
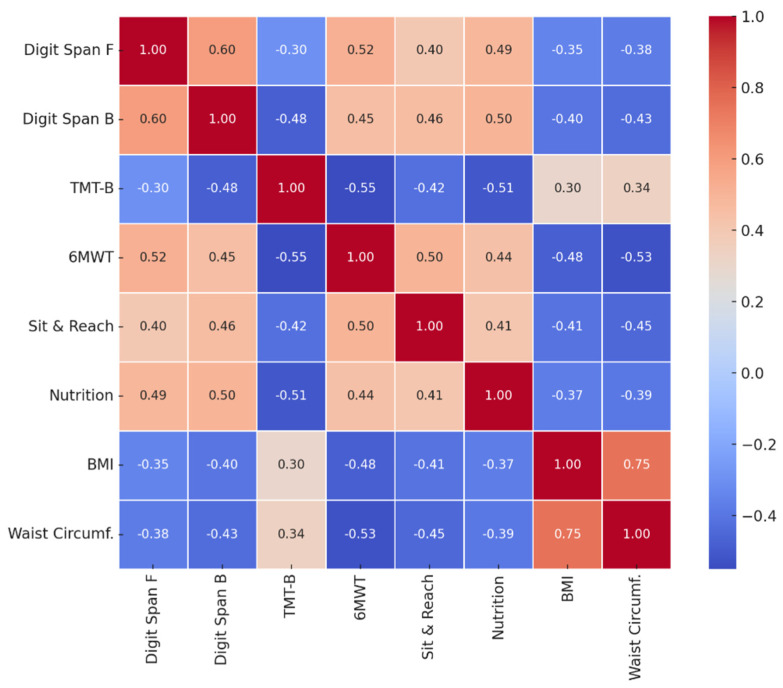
Matrix of correlations.

The Pearson correlation matrix graph (Figure 3) provides a clear visual representation of the relationships between the variables considered in the experimental group post-test study.

Deep red coloring indicates strong positive correlations, meaning that an increase in one variable is associated with an increase in the other. For example, there is a strong positive correlation between Digit Span Forward and Digit Span Backward, suggesting that short-term and working memory capacities are interconnected.

Deep blue coloring represents strong negative correlations, where the increase in one variable is associated with a decrease in the other. For example, Trail Making Test B shows a strong negative correlation with the 6-Minute Walk Test, suggesting that better performances (lower times at TMT-B) are associated with greater physical endurance. Similarly, Waist Circumference is negatively correlated with the 6-Minute Walk Test, indicating that a smaller waist circumference is associated with greater aerobic capacity.

Neutral or intermediate stains represent weak or moderate correlations, which although statistically significant (*p* < 0.05), reflect less marked associations. For example, the relationships between BMI and Nutrition Knowledge Score show a moderate negative correlation, suggesting a link between greater nutritional awareness and better body profile.

## 4. Discussion

The present study proposed an integrated intervention, articulated in nutritional education and conscious, creative and environmental physical activity, conducted in a sample of secondary school students with indicators of overweight and obesity, adopting a quasi-experimental design with an experimental group and control group. The main objective was to evaluate the effectiveness of the intervention in improving cognitive functions, perceived well-being and dietary knowledge. The results obtained show significant improvements in the experimental group compared to the control group in all cognitive tests administered, i.e., Digit Span Forward, Digit Span Backward, Stroop Test and Trail Making Test B, as well as improvements in motor tests such as the 6-Minute Walk Test and the Sit and Reach Test, BMI and abdominal circumference, as well as an increase in the score relating to food knowledge. These data confirm the hypothesis that an integrated educational and motor intervention can positively affect the cognitive abilities of students with problems related to body weight, and are in continuity with what is widely reported in the scientific literature that associates obesity with cognitive deficits, in particular in the field of executive functions, attention and working memory.

First, the improvement observed in the Digit Span Forward and Backward tests, which measure short-term working memory and information manipulation ability, respectively, can be interpreted in light of evidence linking regular physical activity and proper nutrition to increased brain plasticity and improved prefrontal circuit efficiency. Neuroscientific studies have shown that aerobic exercise promotes angiogenesis and neurogenesis in the hippocampus, a key structure for memory, as well as improving functional connectivity in neural networks involved in working memory [45,46]. At the same time, a balanced diet, rich in essential nutrients such as omega-3 fatty acids, antioxidants and vitamins, helps reduce oxidative stress and neurogenic inflammation, factors implicated in cognitive deficits associated with obesity [47]. Thus, the combination of nutrition education and body movement likely resulted in an environment conducive to improved performance on working memory tests, supporting students’ ability to retain and manipulate information relevant to school and daily activities [48].

Secondly, the positive outcome obtained in the Stroop Test, which assesses inhibitory control and selective attention, and in the Trail Making Test B, indicative of cognitive flexibility and the ability to alternate tasks, underlines an improvement in higher-order executive functions. These functions, essential for behavior regulation and problem solving, are often impaired in subjects with excess weight, due to neurobiological alterations such as dopaminergic dysfunction and reduced activity in the prefrontal cortex [49]. Regular physical activity has been shown to increase dopamine and brain-derived neurotrophic factors such as the brain-derived neurotrophic factor (BDNF), which promote synaptic plasticity and prefrontal network function. In addition, the laboratory and dialogical approach of nutritional education, which encouraged critical reflection and active participation, may have stimulated metacognitive and self-control processes, further contributing to the observed improvement of cognitive regulation. This finding aligns with previous research emphasizing the key role of integrating cognitive and emotional stimuli into intervention strategies for adolescents with obesity-related difficulties [50].

The improvement in motor tests, highlighted by the 6-Minute Walk Test and the Sit and Reach Test, confirms not only an increase in aerobic capacity and muscle flexibility, but also a potential facilitating effect on cognitive efficiency. Numerous studies support the hypothesis that improved overall physical condition promotes cerebral perfusion and neuronal metabolic efficiency, thus promoting an optimal environment for cognitive functions [51,52,53]. In addition, the creative and environmental component of the motor pathway, which included activities in natural and urban contexts, helped to stimulate greater body awareness and positive emotional involvement—elements that seem to amplify the beneficial effect of physical activity on cognitive functions [54]. These results are confirmed by studies that underline the role of integrated motor experience as a protective factor against cognitive decline, especially in developmental age groups such as adolescence [55].

Among the most relevant results that emerged from the integrated intervention, the significant improvements found in the anthropometric indicators of BMI and waist circumference in the experimental group compared to the control group are highlighted. These data confirm the effectiveness of the approach adopted in promoting positive changes in the physical and metabolic field among secondary school students with overweight and obesity conditions. In particular, the reduction in BMI suggests a rebalancing of the relationship between weight and height, while the decrease in waist circumference represents a significant signal of reduction in visceral fat, a known risk factor for numerous chronic diseases. These results are particularly important when considered in the light of the young age of the participants, as early intervention is a crucial element in the prevention of obesity in adulthood [56,57].

These findings appear particularly relevant when compared to previous large-scale school-based interventions, such as [58,59], which directly implemented nutritional and physical activity changes but did not detect significant impacts on markers of obesity. In contrast, the present integrated approach yielded measurable improvements in BMI and waist circumference, suggesting that the combination of conscious, creative, and environmental physical activity with nutritional education may overcome some of the limitations observed in earlier interventions.

The integrated approach, based on conscious, creative and environmental physical activity, together with a nutritional education path, seems to have favored greater adherence to good practices of movement and nutrition, activating processes of body and behavioral awareness [60,61]. Although the primary objective of the study was to evaluate the impact of the intervention on cognitive functions, perceived well-being and dietary knowledge, the observed changes in anthropometric parameters represent an added value, suggesting a positive impact also on physical health.

These results support the hypothesis that multidimensional and contextualized interventions, which actively involve students in meaningful and stimulating environments, can generate concrete and measurable impacts also in terms of physical, as well as cognitive and emotional, health [62].

Furthermore, the integrated design of the intervention may have fostered a more intrinsic engagement among students, particularly due to the experiential and playful nature of the physical activities, as well as the dialogical structure of the nutritional education sessions. These aspects are consistent with the Self-Determination Theory [63], which posits that autonomy, competence, and relatedness are key psychological needs that, when fulfilled, enhance motivation and learning outcomes. By promoting autonomy (through creative physical activities), competence (through progressive skill acquisition), and relatedness (through group participation), the program may have supported students’ internal motivation to adopt healthier lifestyles and engage cognitively in the tasks proposed.

Moreover, from an embodied cognition perspective [64], the integration of movement and sensory experiences within natural and urban environments may have contributed to reinforcing cognitive learning by activating multimodal processing channels. Such embodied and situated learning experiences are increasingly recognized in educational neuroscience as powerful facilitators of cognitive and emotional development, particularly in adolescents.

The improvement in food knowledge also suggests that the educational approach, characterized by active, cooperative, and reflective methodologies, enhanced not only factual learning but also critical thinking about lifestyle habits. This supports existing evidence indicating that when nutrition education is delivered through participatory and reflective pedagogies, students are more likely to retain knowledge and transfer it to behavior [65].

A possible explanation for the differential improvements between the experimental and control groups lies in the holistic and integrative nature of the intervention, which moved beyond the mere transmission of information. While traditional school-based programs often rely on didactic methods, this intervention combined physical, cognitive, and relational dimensions, thus producing synergistic effects on students’ overall functioning [66].

Finally, it is worth noting that the observed improvements in both cognitive and physical parameters align with recent meta-analyses that emphasize the importance of multimodal interventions in addressing adolescent obesity and its neurocognitive correlates [67]. However, unlike many previous studies that isolate variables, this study emphasizes the importance of ecological validity and contextual engagement, suggesting that sustainable change may depend more on the meaningfulness of the experience than on its intensity or duration alone.

However, the study has some limitations that suggest a conservative interpretation and point to areas for improvement for future research. Although a randomization at the class level has been adopted, this procedure, aimed at not altering the pre-existing teaching dynamics, may have limited the full randomness in the assignment of participants to groups, thus introducing potential selection biases. The sample size, although adequate for a preliminary survey, is relatively small and limited to a specific school context, reducing the possibility of extending the conclusions to other geographical or cultural realities. In addition, the absence of medium-long term follow-up prevents the persistence of the cognitive, motor and nutritional benefits detected from being evaluated over time. Finally, the complexity of the intervention, although representative of an integrated approach, makes it difficult to isolate the specific effect of each component, suggesting the need for future studies that separately investigate its impact and possible interactions. Despite these limitations, the methodological strengths and the innovativeness of the proposed approach confirm the value of the study as a significant contribution to the promotion of effective school interventions in the prevention and management of the consequences of obesity on cognitive functions in adolescents.

## 5. Conclusions

The relationship between obesity and the impairment of cognitive functions is a topic of increasing relevance within the scientific literature, particularly in light of the exponential rise in cases of overweight and obesity among adolescents and the multidimensional implications of these conditions, not only at the physical level but also at the psychological and cognitive levels. The results of the present study suggest that an integrated intervention, which combines nutritional education with conscious, creative, and environmentally oriented physical activity, can be a valuable tool to promote significant improvements in cognitive functions, perceived well-being, and nutritional knowledge among lower secondary school students with overweight and obesity indicators.

While these findings are promising, caution must be exercised when generalizing them to broader populations. The study was conducted in a specific geographical and cultural context, with a limited sample and a short-term intervention period. These constraints may influence the external validity of the results. Furthermore, the composite nature of the intervention does not allow for a clear distinction of the contribution of each component, which warrants further research.

Nevertheless, the data support the idea that schools should be conceived not only as places for health prevention but also as generative environments of integrated well-being, where disciplinary learning and embodied practices come together to support the holistic development of students. This integrated vision opens new perspectives for the systemic and curricular implementation of educational programs that promote health in a holistic and participatory manner.

In terms of future research directions, it is essential to conduct randomized experimental studies on larger and more diverse samples to enhance the generalizability of findings and to further investigate the neurobiological mechanisms underlying the observed cognitive improvements. Longitudinal designs are also needed to assess the sustainability of the cognitive, motor, and behavioral benefits over time. A more detailed analysis of the individual components of the intervention could help identify their specific contributions and improve the effectiveness of educational strategies.

Finally, recognizing the school as a privileged context for the promotion of integrated health and well-being must translate into a systemic commitment by educational institutions. This entails the definition of curricular guidelines and school policies that align with a pedagogical model which acknowledges the interconnectedness of nutritional, motor, and cognitive dimensions, thereby contributing meaningfully to the prevention and management of the consequences of adolescent obesity.

In conclusion, this study provides concrete evidence for the effectiveness of multidimensional interventions at the lower secondary school level, while acknowledging the limitations that affect the scope of generalization. It supports the adoption of integrated approaches that promote overall adolescent health and offers a foundation for an educational model capable of significantly contributing to the well-being of future generations.

## 6. Practical Implications

The results of this study offer valuable insights for the design and implementation of school-based interventions aimed at promoting both the physical and cognitive well-being of adolescents, especially those affected by overweight and obesity. The integrated approach tested in this study demonstrates high operational feasibility. The proposed physical activities do not require specialized equipment, can be implemented in informal or natural settings, and are based on inclusive, experiential, and active methodologies that engage students physically, emotionally, and cognitively.

The nutritional education component also proved effective in increasing awareness and knowledge through the use of accessible and replicable tools such as food diaries, guided discussions, and participatory reflections. These tools can be easily incorporated into existing curricula and adapted to various educational settings.

From an institutional perspective, this intervention model highlights the importance of integrating physical education, health sciences, and transversal life skills into a cohesive framework. This offers a sustainable and evidence-based strategy for addressing the cognitive, emotional, and behavioral consequences of adolescent obesity. Active student engagement and experiential learning emerged as key drivers of self-efficacy and responsibility, which are essential for achieving long-term behavioral change.

Furthermore, the model may be enriched through partnerships with local communities, health professionals, and families, providing a replicable example of effective educational practice. In this light, schools reaffirm their role not only as places of academic learning but also as strategic environments for promoting holistic adolescent development and well-being.

## Figures and Tables

**Table 1:** Descriptive characteristics of the sample.

Variable	Experimental Group (*n* = 30)	Control Group (*n* = 30)	Total (*N* = 60)
Mean age (years)	12.5 (±0.3)	12.4 (±0.4)	12.5 (±0.3)
Gender (Female/Male)	15/15	14/16	29/31
Mean BMI (kg/m^2^)	23.4 (±2.8)	23.1 (±2.6)	23.3 (±2.7)
Overweight percentage (%)	40%	37%	38.5%
Obesity percentage (%)	30%	27%	28.5%
Students with Law 104/92 certification	1	2	3
Regular school attendance (%)	100%	100%	100%

Note: values in parentheses indicate standard deviation (SD) for continuous variables.

**Table 3:** Summary of confirmatory factor analysis fit indices.

Model Domain	χ^2^ (*df*)	χ^2^/*df*	CFI	TLI	RMSEA (90% CI)	SRMR	KMO	Bartlett’s Test (*p*)
Cognitive Function	52.78 (28)	1.88	0.94	0.92	0.065 [0.043–0.082]	0.048	0.79	<0.001
Physical and Anthropometric	24.66 (14)	1.76	0.96	0.94	0.059 [0.034–0.088]	0.041	0.75	<0.001
Nutritional Knowledge	3.21 (2)	1.60	0.99	0.98	0.042 [0.000–0.112]	0.022	0.84	<0.001
Overall 3-Factor Model	84.65 (45)	1.88	0.95	0.93	0.064 [0.048–0.078]	0.051	0.81	<0.001

**Table 4:** Descriptive statistics of pre- and post-test measurements.

Variable	Group	Pre-Test M	Pre-Test SD	Post-Test M	Post-Test SD	Δ (Post − Pre) M
Digit Span Forward	Experimental	5.4	0.7	6.2	0.7	+0.8
	Control	5.3	0.6	5.2	0.6	−0.1
Digit Span Backward	Experimental	4.1	0.7	4.75	0.7	+0.65
	Control	4.0	0.65	3.95	0.65	−0.05
Stroop Test (s)	Experimental	55.4	5.8	49.2	5.5	−6.2
	Control	54.9	6.0	54.8	6.1	−0.1
Trail Making Test B (s)	Experimental	98.0	10.2	89.5	9.8	−8.5
	Control	97.2	9.9	96.7	9.7	−0.5
6-Minute Walk Test (m)	Experimental	590.0	35.0	632.4	34.8	+42.4
	Control	588.5	33.5	598.7	32.8	+10.2
Sit and Reach Test (cm)	Experimental	18.5	3.4	21.6	3.3	+3.1
	Control	18.2	3.6	18.9	3.5	+0.7
Nutrition Knowledge Score	Experimental	14.8	1.7	17.4	1.6	+2.6
	Control	14.7	1.8	14.6	1.9	−0.1
BMI (kg/m^2^)	Experimental	21.1	2.2	20.3	2.0	−0.8
	Control	21.2	2.3	21.2	2.3	0.0
Weight (kg)	Experimental	49.4	5.15	47.5	4.68	−1.9
	Control	49.6	5.38	49.6	5.38	0.0
Waist Circumference (cm)	Experimental	79.3	4.5	74.8	4.3	−4.5
	Control	78.7	4.8	78.2	4.7	−0.5

**Table 5:** ANCOVA results.

Variable	F(1,57)	*p*-Value	Partial η^2^
Digit Span Forward	14.86	<0.001	0.21
Digit Span Backward	12.45	0.001	0.18
Stroop Test (s)	16.92	<0.001	0.23
Trail Making Test B (s)	11.08	0.001	0.16
6-Minute Walk Test (m)	13.74	<0.001	0.19
Sit and Reach Test (cm)	10.33	0.002	0.15
Nutrition Knowledge Score	18.60	<0.001	0.25
BMI (kg/m^2^)	6.40	0.05	0.10
Waist Circumference (cm)	8.30	0.01	0.12
Weight (kg)	7.10	0.01	0.11

**Table 6:** Pearson correlations between post-test measures (experimental group).

Variable 1	Variable 2	Pearson’s r	*p*-Value
Digit Span Forward	6-Minute Walk Test	0.52	0.003
Digit Span Forward	Nutrition Knowledge Score	0.49	0.005
Digit Span Backward	Sit and Reach Test	0.46	0.008
Digit Span Backward	Nutrition Knowledge Score	0.50	0.004
Trail Making Test B	6-Minute Walk Test	−0.55	0.002
Trail Making Test B	Digit Span Backward	−0.48	0.006
Nutrition Knowledge	Trail Making Test B	−0.51	0.004
BMI (kg/m^2^)	6-Minute Walk Test	−0.48	0.006
BMI (kg/m^2^)	Sit and Reach Test	−0.41	0.015
BMI (kg/m^2^)	Nutrition Knowledge Score	−0.37	0.028
Waist Circumference	6-Minute Walk Test	−0.53	0.003
Waist Circumference	Sit and Reach Test	−0.45	0.009
Waist Circumference	Nutrition Knowledge Score	−0.39	0.022

## Data Availability

The data presented in this study are available on request from the corresponding author. The data are not publicly available due to privacy restrictions.
